# Notes and News

**Published:** 1890-08-16

**Authors:** 


### Notes and News.

Collected and Communicated.

Cardinal Newman is dead. He was an Anglican first, and then a Roman Catholic, but always an Englishman. With some peculiarities of intellect he was a man of the deepest earnestness of character, and the most devoted loyalty to conscience. In so far as his ecclesiastical position was concerned, he lived in an atmosphere unfamiliar to most of his fellow-countrymen, and unsuited to the national character. But in that he was a firm-willed, self-denying, and duty-loving man; he has given to the world an example which everyone may follow. All England respected him, and all England will mourn for him.

The committee of the Zenana Medical College, St. George's Road, S.W., have much pleasure in announcing that T.R.H. the Duke and Duchess of Connaught have consented to become presidents and the Duke of Clarence and Avondale patron. The daughter of an Armenian pastor has entered the college as a student. Next session opens on October 1st.

Oban is to have its Cottage Hospital, and the architect is Mr. Robert Mortimer, of Westminster. The building will have a south-westerly exposure, and its general form of plan will be that of the letter L, to one arm of which one of the wards will form an additional projection on the ground floor. The external appearance indicates that when completed the building will form an attractive addition to the town, giving one the idea of its being a decidedly pretty villa residence. The funds have been provided by an anonymous donor, so tourists need not be afraid to approach the favourite Scotch watering-place.

The following preventive measures against cholera have been approved by the French doctors, &c., and may be useful to those who are going to infected districts for their holidays:—

"Sanitary precautions are indispensable during a time of epidemic. It is necessary to be warmly clothed and to avoid any risk of catching a cold.

"Wear a flannel belt next the skin.

"The abuse of wine and alcoholic liquors, the excessive use of iced water, raw fruit, and all uncooked food, as well as excesses of every kind, should be avoided.

"It is essential to use nothing but clear water well filtered, and, if possible, boiled, and then aërated after cooling. Natural mineral waters are also very good to use."

Mr. Henry Irving and Mrs. Frederick Hawkins last week visited the Lowestoft Hospital, and were shown round by the matron and house surgeon. On leaving, Mr. Irving wrote a cheque for the hospital, and also signed his name in the visitors' book. On the same afternoon Mrs. Beauchamp Wildman sang in the wards. The patients at seaside hospitals have cause to rejoice in the holiday season.

The long list of scandals at Hope Hospital is to have another addition; let us hope that at last the root of the evil will be reached, and that this handsome infirmary will become a credit to its committee. The Hope Hospital, Eccles, was built eight years ago on the pavilion system; it is a union infirmary. During these eight years there have been two Government inquiries concerning the management of the institution; there have been six matrons and three medical superintendents. Our readers will remember the last scandal of just about a year ago; a scandal with a small outward cause—rats in the matron's bed—but which undoubtedly pointed to internal maladministration. It caused a great sensation at the time, but the conclusion of the inquiry was eminently unsatisfactory.

Now Dr. Watson, assistant medical officer, has preferred certain charges against Dr. Conry, medical superintendent. Mr. Mottram, one of the guardians, said at a late meeting he hoped that the guardians would keep clear of the question altogether, and let it be fought out between the men themselves. He moved a resolution that the guardians have no witnesses to call. The Mayor presumed that the guardians had only one object in view—to get at the truth. He did not care which doctor was right and which was wrong, but what he did care for was the interests of the institution, and he trusted that the inquiry would be a searching one, reaching the management itself. By passing the proposed resolution the inquiry would be simply burked, and therefore he protested against it. He hoped the guardians would not allow themselves to be handicapped in that way, and be left at liberty to call witnesses. The enlightened Board, however, refused to uphold the Mayor, and the result is that the inquiry is "burked" and left to be a personal quarrel. The ratepayers of Salford need rousing.

Several papers are falling foul of "church parades" just now; the secretary of the Westminster Hospital says the bands annoy the patients and nurses; the *World* says: "These Sunday parades are becoming a public scandal. With noisy bands and vulgar banners, with men, women, and children dressed up in fantastic garb, with clowns on stilts, carrying their collecting-boxes to the balconies and upper windows of houses, these processions pass through the various streets by the most circuitous route on their way to the church of St. Pancras or St. Marylebone, attracting great crowds and causing much disturbance to the peace and quietude of the residents. The same performance is repeated after the service at church has concluded, and there have been not a few Sundays this season devoted to this kind of buffoonery." Just a little care on the part of the organisers of these parades and these complaints might be avoided. The friendly societies are unfortunate in one of their supporters. A paper, which shall be nameless, wrote: "Nurses who take a little nap at noon are also fretted by these demonstrations of the brutal democracy. Some of them were wakened twice in their mid-day siesta, &c., &c. Hence let no processions of working men be allowed to go near Westminster Hospital," and other stupid remarks in the same mistaken vein. The nurses who were awaken were nurses who, for the sake of working men, turn night into day. It was no little siesta, but a hard-earned sleep after a night's work, from which they were awakened.

The following vacancies are announced this week, the amount of the salary in each case being given after the name of the institution:—

**Resident Medical Officers.**

Torbay Hospital and Dispensary, Junior House Surgeon and Dispenser—board, etc.

Ripon Hospital and Dispensary, House Surgeon and Dispenser—£60, board, etc.

**Non-Resident Medical Officers.**

Children's Hospital, Manchester, Medical Officer—£180.

Celbridge Union, Medical Officer—£115.

---

On the 5th instant, the Stephen Cottage Hospital at Dufftown was opened by the Duke of Fife, who, accompanied by his suite, arrived about one o'clock. The weather was magnificent, and a large crowd congregated to witness the interesting event. The town was profusely decorated in honour of the occasion. A procession started from the post office, and marched to the hospital. After an inspection of the buildings his Grace emerged to the door of the main entrance where he formally declared the hospital open. The hospital is given to the town by Sir George Stephen, a native of Dufftown, who has made a fortune in Montreal. The building is from plans by Messrs. A. and W. Reid, and is a one-storey building, with two large wards and the usual offices.

---

A large number of visitors attended the annual meeting of the Royal Sea Bathing Infirmary at Margate. A short service was held in the Infirmary Church, then the wards were visited, and then the report was submitted. The resident surgeon reports that the work done during the past year is of a most satisfactory and encouraging character, the percentage of *cured* cases having reached 45·18 per cent., which is a very high one when the nature of the disease in all its stages is taken into consideration. The falling-off in funds was sadly referred to, but all looked forward to great deeds at the centenary celebration next year. The local papers publish a portrait and memoir of Sir Erasmus Wilson, one of the first supporters of the institution.

---

The outbreak of diphtheria at Paddington still remains somewhat of a mystery, though the inquiries of the Sanitary Committee have been searching. The reports of Dr. Stevenson, the Medical Officer of Health, reveal that for the last four years there has been an undue mortality in Paddington from this cause. In 1886-7 there were 28 deaths from diphtheria. One death less was registered in 1887-8. The following year showed the greatly increased total of 75. The 1889-90 report gives 39 deaths. By virtue of the Infectious Disease (Notification) Act the vestry possesses the power to render the notification of measles compulsory, and, as diphtheria is a common sequence to measles, it has been decided that, as information of the presence of the latter may enable a check to be placed on the spread of the former, the powers shall be enforced. As a means of general precaution this is all that is recommended to be done until after the recess. Notices in respect of defective and combined drainage are to be served on the owners of Nos. 142, 144, 146, and 148, Westbourne Terrace. Not only has a second case of diphtheria occurred at the house No. 144, but the drainage of the adjoining houses has been found to be in a very bad state. The existing system of combined drainage at the rear of the premises is accordingly to be abolished, and there is to be forthwith constructed an extension of the existing sewer in Orsett Terrace to a point opposite the entrance to the Great Western Railway store-yard in Westbourne Terrace at a cost of over £400. The tenants of these houses have protested against this order, and hope to get the decision reversed.

The Salford Day Nursery issues a very artistic and interesting little report—evidently meant to appeal to the susceptibilities of the gentler sex. But in spite of the poetry and the tinted paper, it is a practical little pamphlet telling of good work done in the true spirit. There is a balance-sheet and a condensed account of the annual meeting; also a list of annual subscribers. May all subsequent reports of this charity tell as pleasant a tale in as pretty a manner!

---

The Charity Organization Society is to be congratulated on its victory in the case of the bogus institution of William Harper Bradshaw. The defendant, it was said, was the promoter of a seaside home for poor children at Southend, and that he had solicited contributions with specious misrepresentations. A building known as "The Glen," pictorially represented as the Children's Home, with carriage drives and sea view, turned out to be shut up and unoccupied, and it was said that although in recent appeals for help allusion was made to special arrangements with railway and boat companies for the cheap conveyance of children, there was nothing to warrant such a statement at the present time, and that it was an entirely obsolete reference to a former institution with which the defendant was connected. With regard to this, Mr. Wontner, who prosecuted, said defendant could not get on with a committee, that there had been no proper account of the funds received, and that in point of fact there was only some pretence made to carry on a charity for Bradshaw to put money in his own pocket. Sir John Bridge said a fine would be no adequate punishment for such a fraud as this, calculated as it was to stop the flow of charity. It was pleaded that defendant was ignorant of the law, but he seemed ignorant of the first rudiments of morality. Convicting him as a rogue and vagabond, he sentenced him to three months' hard labour. It is a pity Sir James Walker, who was Bradshaw's latest victim, had not provided himself with the Charity Organization "Cautionary Information Card," on which Bradshaw has been pilloried for many a long year.

---

The annual report of Manchester Royal Infirmary, which was submitted at the late meeting, stated that the total number of cases treated during the last twelve months was 46,205. In the year ending June 1885, it was 30,726, and in that ending June, 1880, it was 23,596. These figures afforded convincing proof of the great and beneficent work which was being done in connection with the infirmary in alleviating the suffering of the sick poor in the district. During the year, through the kindness of the committee of the Cotton Districts Convalescent Fund, eighty of the in-patients had been sent to the hospital at Southport or to the Devonshire Hospital, Buxton. The report went on to add that in January last the medical staff urged upon the Board the necessity of increasing the number of beds at the infirmary, and of making certain alterations and additions to the wards. They stated that there was a great and increasing pressure upon the existing accommodation; that delay frequently took place in the admission of suitable cases; that patients were sometimes discharged sooner than under other circumstances they would be; and that a larger number of patients per bed are treated at the infirmary than at any other general hospital in the United Kingdom. The income of the infirmary and of the Convalescent Hospital at Cheadle for the year had been:—Royal Infirmary, £21,115; Convalescent Hospital, £2,534. The accounts of the Monsall Fever Hospital showed that its income during the past year had amounted to £14,123. The legacies amounted to £24,812 5s. 3d. The total expenditure had been:—Infirmary, £17,125; Convalescent Hospital, £5,193; and Monsall, £10,416. The Royal Infirmary contained 298 beds; the Convalescent Hospital, Cheadle, 136; Monsall Fever Hospital, 306; Royal Lunatic Hospital and its branches, 260; a total of 1,000 beds.

